# Albright hereditary osteodystrophy: Delay in the diagnosis of a rare disorder due to restricted medical services

**DOI:** 10.1002/ccr3.6841

**Published:** 2023-01-16

**Authors:** Sahar Noor, Nasrin Hakimzada, Nijatullah Safi, Sultan Mahmood Alikozai, Abdul Jamil Rasooli, Tooryalai Jalalzai, Qais Siddiqui, Ahmad Jalil Sestani, Najla Nasir, Sarah Noor, Ahmed Maseh Haidary, Saifullah Khalid

**Affiliations:** ^1^ Department of Paediatric Medicine French Medical Institute for Mothers and Children (FMIC) Kabul Kabul Afghanistan; ^2^ Department of Medicine Rabia Balkhi Hospital Kabul Afghanistan; ^3^ Department of Oncology Ali Abad Hospital Kabul Afghanistan; ^4^ Department of Pathology French Medical Institute for Mothers and Children (FMIC) Kabul Kabul Afghanistan; ^5^ Department of Radiology Jumhoriat Hospital Kabul Afghanistan

**Keywords:** Albright hereditary osteodystrophy, brachydactyly, hypocalcaemia, pseudohypoparathyrodism

## Abstract

A teenage Afghan girl presented with seizure. Clinical features and laboratory investigations revealed elevated serum parathormone, high phosphate levels with low serum calcium. In third‐world countries, diagnosis of rare disorders, such as Albright hereditary osteodystrophy (AHO), can usually be delayed due to scarcity of standard medical and diagnostic services.

## 1 BACKGROUND

Albright hereditary osteodystrophy (AHO), a rare clinical entity, is inherited in autosomal dominant or autosomal recessive pattern.^1^ AHO is commonly observed in pseudohypoparathyrodism (PHP) type 1A, type 1C, and pseudopseudohypoparathyrodism (PPHP) while it is rarely diagnosed in patient with PHP1B, but not in PHP2.^1^

PHP occurs as a result of genetic as well as epigenetic defects at *guanine nucleotide binding protein alpha‐stimulating activity polypeptide* (*GNAS*) gene locus. The *GNAS* gene is located on the telomeric end of the long arm of chromosome 20 (20q13.2‐20q13.3).^2^ PHP1A and PHP1C occur as a result of maternally inherited inactivating *GNAS* mutations that clinically manifest with AHO phenotype including parathyroid hormonal resistance.^2^ In contrast, patients with paternally inherited *GNAS* mutations present with the AHO phenotype alone, without parathyroid hormonal resistance (PPHP).^2^ Similarly, PHP1B results due to methylation abnormalities of *GNAS*.^2^

The disease has a wide range of signs and symptoms, but the characteristic phenotypic features of AHO are short stature, round face, short neck, subcutaneous ossifications, and brachydactyly of long bones including metatarsal and metacarpal bones, especially of fourth and fifth digits. The patients also have developmental delay, dental defects, and convulsions.^3^

Here we present a case of AHO in which there was significant delay in the diagnosis of a rare disorder due to restricted medical services, in spite of very obvious clinical as well as biochemical features.

## 2 CASE PRESENTATION

A 16‐year‐old Afghan girl was referred to the French Medical Institute for Mothers and Children (FMIC) Kabul, Afghanistan, by provincial hospital, where she had presented with history of abnormal movement of limbs and eyes since early childhood which was poorly controlled. Family history was unremarkable and there was no consanguinity of her parents.

**FIGURE 1:**
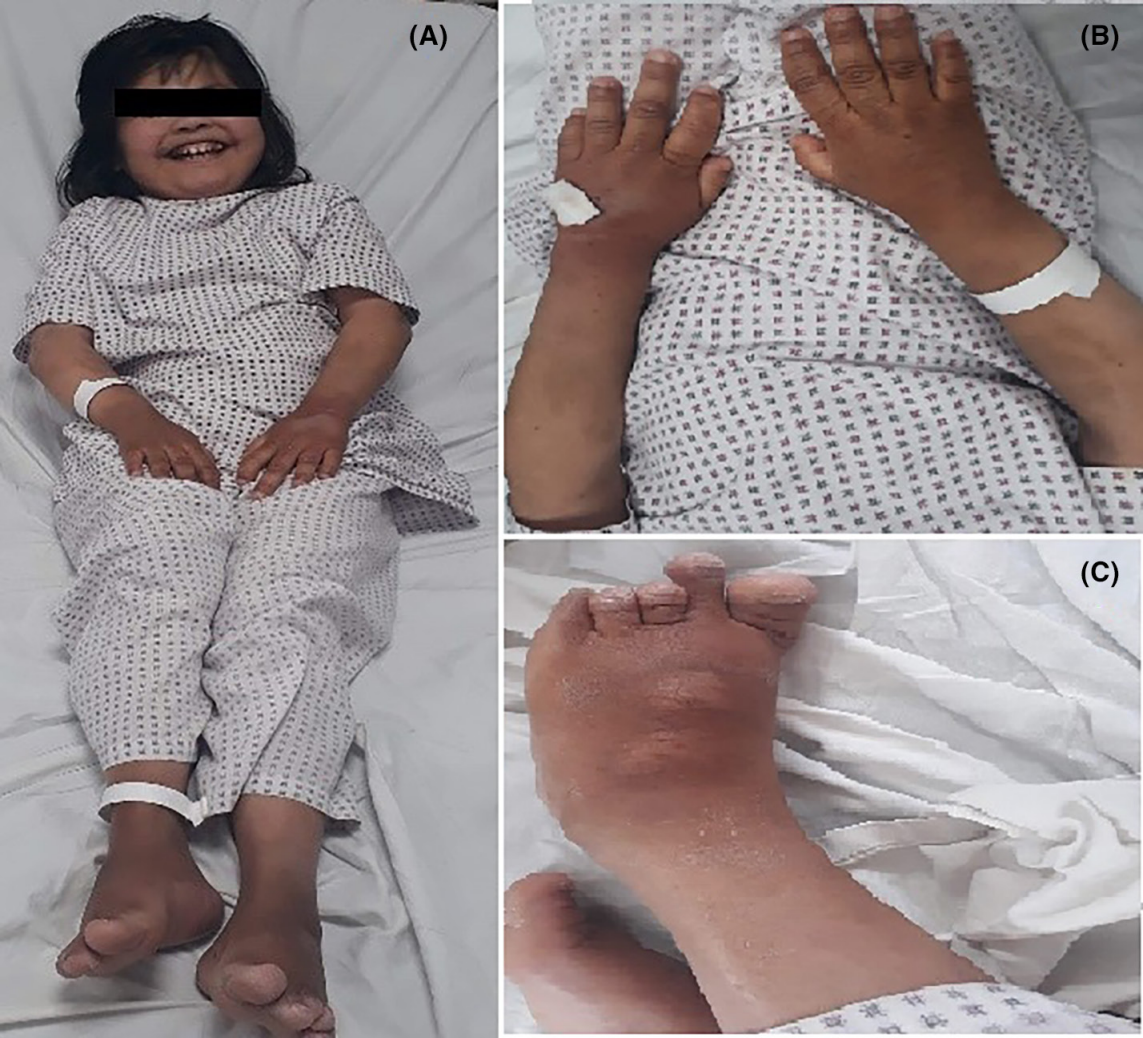
(A) Patient with AHO having short stature, short neck, and round face, (B) Bradydactyly of metacarpals and (C) Bradydactyly of metatarsals.

General physical examination revealed short stature, round face, depressed short neck as shown in Figure 1A. Other features included sparse hair on scalp, depressed nasal bridge, coarse face and skin texture, dental carries, and poor periodontal status. She had brachydactyly of fourth and fifth digits of hands and feet as shown in Figure 1B,C. Patient was obese with body mass index (BMI) of 35.3 for a height 133 cm and her weight was 62 kg.

As shown in the Table 1, laboratory investigations revealed low serum calcium with a value of 4.6 mg/dL, high serum phosphate with a value of 7.6 mg/dL, and parathyroid hormone (PTH) of 85 pg/mL. She also had vitamin D deficiency with a value of 5 ng/mL, while thyroid‐stimulating hormone (TSH) was normal. Computed tomography (CT) scan of brain reveals calcification involving basal ganglia, periventricular region, and cerebellar hemispheres.

**TABLE 1:** Laboratory investigations

Laboratory investigations		
Parameters	Results	Normal values
PTH	85 pg/mL	10–55 pg/mL
Serum Vitamin D	5 ng/mL	25–80 ng/mL
**Thyroid function test**		
TSH	2.1 mlU/L	1.3–2.35mlU/L
Free T3	3.83 pg/mL	2.67–4.62 pg/mL
Free T4	1.3 ng/dL	0.8–1.9 ng/dL
**Serum electrolytes**		
Sodium	140 mEq/L	136–146 mEq/L
Potassium	3.5 mEq/L	3.5–4.5 mEq/L
Chloride	98 mmol/L	96–106 mmol/L
Calcium	4.6 mg/dL	9–11 mg/dL
Magnesium	1.8 mEq/L	1.3–2.1 mEq/L
Phosphate	7.6 mg/dL	3.0–4.5 mg/dL

Abbreviations: L, liter; mEq., milliequivalent; ml, milliliter; pg., pictogram; PTH, parathyroid hormone; TSH, thyroid‐stimulating hormone.

## 3 DISCUSSION AND LITERATURE REVIEW

Albright et al. were the first to investigate and report the first cases of hypoparathyroidism associated with seizures, hypocalcaemia, and hyperphosphatemia.^4^ To their surprise, repeated injections of bovine parathyroid extracts to the patient failed to alter the serum calcium and phosphate levels leading to the impression of PHP.^4^ Additional features included short stocky build, round face, cutaneous ossification, and metacarpophalangeal abnormalities.^4^ The abovementioned constellation of features was therefore given the label of AHO.^4^ Occasionally, patients presented with typical features of AHO but normal serum calcium and phosphate.^5^ Such patients with similar clinical features but the absence of PTH resistance, were later labeled by Albright et al. as PPHP.^5^

PHP occurs due to heterozygous inactivating mutation in the *GNAS* gene that codes α‐subunit of the stimulatory G protein.^6^ *GNAS* gene is located on the telomeric end of the long arm of chromosome 20.^7^ PHP1A and PHP1C result due to maternally inherited inactivating *GNAS* mutations, which manifest with AHO phenotype plus hormonal resistance, in which PHP1A is characterized by inability of the body to respond appropriately to parathyroid hormone, resulting in hypocalcemia, increased serum parathyroid hormone concentration, insensitivity to biologic activity of parathyroid hormone, and hyperphosphatemia.^3^ AHO when associated with resistance to parathormone, is called PHP1C.^3^ Its incidence is 1/100000, twice as frequent in females as in males.^2^ In contrast, patients with paternally inherited *GNAS* mutations present with the AHO phenotype alone without hormonal resistance (PPHP).^3^ PHP1B results due to epigenetic methylation abnormalities of *GNAS*.^2^

PHP is a term that refers to a heterogeneous group of disorders where the most common feature is the resistance to parathyroid hormone.^6^, ^7^ Most of the patients have hypocalcemia and hyperphosphatemia despite elevated levels of parathyroid hormone in plasma. Associated PTH resistance and chronic hypocalcemia and hyperphosphatemia can lead to calcifications in the basal ganglia and subcortical white matter, and consequent neuropsychiatric disorders, movement disorders, and parkinsonism.^8^, ^9^

Patients have increased risk of carpal tunnel syndrome, sleep apnea, reduced insulin sensitivity with impaired glucose tolerance, and spinal stenosis leading to paraparesis of the lower extremities.^10^, ^11^ Other complications such as obstructive sleep apnea, neuropsychiatric disorders, seizures, and cataract have also been reported, but despite these complications, the life expectancy is normal in patients with AHO.^4^, ^12^ Since hypocalcemia and hyperphosphatemia leads to many complications of the disorder, prompt diagnosis can save life and improve life quality.^13^

AHO is a metabolic disorder with obvious clinical and biochemical features. The clinical findings of AHO in our patient that have been described in our report would be a significant contribution to the available literature.^13^

Although significant work has been done to elaborate on the diagnosis and management of AHO, still further large‐scale studies are required to further facilitate a better understanding of the disease.^14^ This would allow for the development of clinical guidelines for early diagnosis and management of such patients. Similarly, a multidisciplinary management approach is essential with the involvement of clinical psychologist, endocrinologist, developmental pediatrician, dentist, and nutritionist, to deal with every aspect of the disorder.^13^

## 4 CONCLUSON

We presented a case of AHO, in which the diagnosis was significantly delayed due to unavailability of standard medical as well as laboratory diagnostic services in Afghanistan. Inherited syndromes such as the one reported in current case report, if diagnosed early, can help to prevent serious and life‐threatening complications.

## AUTHOR CONTRIBUTIONS

**Sahar Noor:** Conceptualization; data curation; investigation; methodology; writing – original draft; writing – review and editing. **Nasrin Hakimzada:** Resources; software. **Nijatullah Safi:** Conceptualization; supervision; validation. **Sultan Mahmood Alikozai:** Conceptualization; resources; software; supervision; validation; writing – original draft. **Abdul Jamil Rasooli:** Conceptualization; investigation; methodology; resources; supervision; visualization; writing – original draft; writing – review and editing. **Tooryalai Jalalzai:** Supervision; validation. **Qais Siddiqui:** Supervision; validation. **Najla Nasir:** Data curation; formal analysis; supervision; validation. **Sarah Noor:** Resources; software; supervision; validation; writing – original draft; writing – review and editing. **Ahmed Maseh Haidary:** Conceptualization; methodology; validation; writing – original draft; writing – review and editing. **Saifullah Khalid:** Formal analysis; validation; visualization.

## FUNDING INFORMATION

The authors received no funding for current writing.

## CONFLICT OF INTEREST

The authors declare to have no competing interests.

## ETHICAL APPROVAL AND CONSENT TO PARTICIPATE

Ethical approval was acquired from the hospital's ethical review committee. Informed consent was obtained from the patient's legal guardian (father) for participation in the current case report.

## CONSENT

Written informed consent was obtained from the patient's legal guardian for publication of this case report and any accompanying images. A copy of the written consent shall be made available for review by the Editor‐in‐Chief of this journal, upon reasonable request.

## Data Availability

All generated data is included in this article.
